# Unusual Distribution of the Arteries

**Published:** 1830

**Authors:** 


4. *Unusual Distribution of the Arteries.*—In a subject which lately came under our observation, the two carotid arteries and the right subclavian arose from the aorta by a common trunk. Just above the upper extremity of the sternum, the carotids separated at an acute angle, and proceeded to their usual position by the side of the trachea. About a quarter of an inch from this angle, the right carotid gave off at a right angle, the right subclavian which under the protection of the clavicle pursued its usual course to the axilla. Under the sternal portion of the sterno-cleido mastoideus, the internal mammary artery was given off, and proceeded between the anterior and middle scaleni muscles to the place of its distribution, and immediately opposite the inferior thyroid arose. F.

5. *To the Editors of the Western Medical Journal.*

GENTLEMEN,

In the next number of your Journal, you will probably confer a benefit on practitioners, and the community, by stating, what, from repeated experience, I have found to be a fact, that exposing a plaster of Cantharides, for a minute or two, to the steam of boiling water, effectually neutralizes the virus which causes *Strangury*, without diminishing the vesicating power of the fly. This was suggested to me by a young practitioner in Philadelphia about three years ago. I regret that his name is not remembered.

A patient of mine has also recently taught me, that, covering the plaster with silk paper saturated in vinegar, is a certain preventative of the above painful effect of the cantharides.

Respectfully,

E. A. ATLEY.

